# Targeting mTOR pathway: A new concept in cancer therapy

**DOI:** 10.4103/0971-5851.76197

**Published:** 2010

**Authors:** S. H. Advani

**Affiliations:** *Jaslok Hospital and Research Centre, Mumbai, Maharashtra, India*

**Keywords:** *Angiogenesis*, *bioenergetics*, *everolimus*

## Abstract

This article highlights the current knowledge of mTOR biology and provides new insights into the role of mTOR in different cancers. An active mTOR coordinates a response in cell growth directly through its effects on cell cycle regulators and indirectly by sustaining nutrient supply into the cell through the production of nutrient transporters and also through the promotion of angiogenesis. A primary way that mTOR exerts its regulatory effects on cell proliferation is by controlling the production of cyclin D1. mTOR increases the translation of hypoxia-inducible factor 1 (HIF-1)/HIF-2. The HIF transcription factors drive the expression of hypoxic stress response genes, including angiogenic growth factors such as vascular endothelial growth factor (VEGF), platelet-derived growth factor β (PDGF-β), and transforming growth factor a (TGF-α). mTOR also increases the surface expression of nutrient transporters proteins. An increase in these proteins results in greater uptake of amino acids and other nutrients by the cell leading to adequate nutrient support to abnormal cell growth and survival. There is also emerging evidence that mTOR activation may play a role in promoting cell survival through the activation of antiapoptotic proteins that contribute to tumor progression. Given that the mTOR pathway is deregulated in a number of cancers, it is anticipated that mTOR inhibitors will have broad therapeutic application across many tumor types. Until now, no treatment demonstrated Phase III evidence after disease progression on an initial VEGF-targeted therapy in advanced renal cell carcinoma. Everolimus is the first and only therapy with Phase III evidence after failure of VEGF-targeted therapy. Everolimus is a once-daily, oral inhibitor of mTOR (mammalian target of rapamycin) indicated for the treatment of advanced renal cell carcinoma in patients, whose disease has progressed on or after treatment with VEGF-targeted therapy.

## INTRODUCTION

The mammalian target of rapamycin (mTOR) pathway is a crucial regulator of cell growth and proliferation and research into this area has revealed that mTOR dysregulation has a key role to play in various cancers. mTOR appears to play a central role in signaling caused by nutrients and mitogens such as growth factors to regulate translation. The understanding of the science behind mTOR’s role as a regulator of many cell processes and its potential as a therapeutic target has opened up treatment possibilities in several types of cancer. This article highlights the current knowledge of mTOR biology and provides new insights into the role of mTOR in different cancers.

mTOR is a 290 kDa serine–threonine kinase that regulates both cell growth and cell cycle progression through its ability to integrate signals from nutrient and growth factor stimuli. mTOR, a member of the phosphatidylinositol 3-kinase (PI3K)-kinase-related kinase (PIKK) superfamily, is composed of 2549 amino acids that are grouped into highly conserved domains. The mTOR is an intracellular kinase that controls the production of proteins through effects on the machinery of mRNA translation. These proteins include important components of several processes critical to cell metabolism, cell growth, cell division, and responses to cellular stresses such as hypoxia or DNA damage.[1,2]

mTOR senses the growth conditions within the cellular environment and helps the cells respond to changes in this environment. An active mTOR coordinates a response in cell growth directly through its effects on cell cycle regulators and indirectly by sustaining nutrient supply into the cell through the production of nutrient transporters and into the cell’s environment through the promotion of angiogenesis. The activation of mTOR signifies a decision point that takes into account the availability of the basic materials required for cell growth (e.g., amino acids, glucose, energy) and the growth-regulating signals from other cells and tissues (e.g., hormones, growth factors) while monitoring conditions of cellular stress (e.g., hypoxia, DNA damage, heat shock, external pH, osmotic stress, oxidative stress). In this manner, the cell is protected from signals outside the cell to grow and proliferate originating when the supply of nutrients and energy inside the cell are not sufficient to support the effort.[3]

## ROLE OF mTOR IN CANCER CELL GROWTH AND PROLIFERATION

Upstream in the growth-promoting pathways are critical molecules that converge on mTOR, which are often deregulated in some manner in cancer. Several mutations found in cancer produce inappropriate signals that activate the mTOR switch, driving the growth and proliferation of the cancer cell.

mTOR senses the availability of nutrients [e.g., adenosine triphosphate (ATP), glucose, amino acids, cholesterol, and iron] and consolidates this information with growth factor signaling through the PI3K/Akt/tuberous sclerosis complex (TSC) pathway.[4] An activated mTOR modulates the rate of protein synthesis for select mRNAs by activating the translational proteins S6 kinase (S6K) and 4E-binding protein 1 (4E-BP1).[1] mTOR activation increases downstream effectors that stimulate cell growth and angiogenesis and regulate cellular metabolism.

A primary way that mTOR exerts its regulatory effects on cell proliferation is by controlling the production of cyclin D1. Cyclins are proteins that regulate the activity of enzymes called cyclin-dependent kinases (CDKs) through the critical G1–S restriction point of the cell cycle, which in turn regulate the passage of cells. Recently, cyclin D1 has been shown to play a role in gene transcription, cell metabolism, and cell migration.

Cyclin D1 overexpression had been associated with a number of cancers including breast cancer, colon cancer, prostate cancer, lymphoma, and melanoma.[5,6]

Mutations in tumor sclerosis complex (TSC1 or TSC2) can also lead to overactivation of mTOR. This overactivation causes unregulated cell proliferation and multisystem tumors in patients with TSC. Increased mTOR activation, as evidenced by hyperphosphorylation of downstream signaling proteins, has been observed in TSC related lesions.[7,8]

## ROLE OF mTOR IN ANGIOGENESIS

mTOR plays a key role in angiogenesis, i.e., the formation of new blood vessels to provide oxygen and nutrients to growing and dividing cells. mTOR increases the translation of hypoxia-inducible factor 1 (HIF-1)/hypoxia-inducible factor 2 (HIF- 2).[9] The HIF transcription factors drive the expression of hypoxic stress response genes, including angiogenic growth factors such as vascular endothelial growth factor (VEGF), platelet-derived growth factor β (PDGF-β), and transforming growth factor-α (TGF-α).[10]

Increased levels of HIF-1α and HIF-1β have been shown to correlate with increased mortality in a number of tumor types, including cervical cancer, breast cancer, non-small-cell lung cancer, ovarian cancer, head and neck cancer, and gastrointestinal stromal tumors.[11] In addition, loss of the von Hippel-Lindau (VHL) protein, which also results in increased levels of HIF-1α, is a primary cause of many cases of renal cell carcinoma (RCC), and has been implicated in pancreatic cancer and neuroendocrine tumors (NETs) as well.[12]

## ROLE OF mTOR IN CELL METABOLISM (BIOENERGETICS)

Cells have a complex sensing system designed to ensure that they do not undergo periods of growth unless adequate levels of nutrients are available to produce the energy necessary to support that growth. The rapidly expanding field of study dedicated to understanding how living systems obtain and utilize energy is known as bioenergetics.

Bioenergetic research has shown that mTOR plays a key role in regulating cell metabolism.[4,13] mTOR increases the surface expression of nutrient transporter proteins. An increase in these proteins results in greater uptake of amino acids and other nutrients by the cell, leading to adequate nutrient support to abnormal cell growth and survival.[13]

Additionally, abnormally activated mTOR may give cancer cells a competitive growth advantage by increasing production of the core enzymes necessary for glycolysis, which enables cancer cells to survive and grow even under hypoxic conditions.[14]

## ROLE OF mTOR IN CELL SURVIVAL

There are several lines of evidence that mTOR activity plays a role in cell survival. Majority of this research has revealed that mTOR inhibition increases sensitivity to cell death pathways; however, there is also emerging evidence that mTOR activation may play a role in promoting cell survival through the activation of antiapoptotic proteins that contribute to tumor progression.[15]

**Figure 1:**
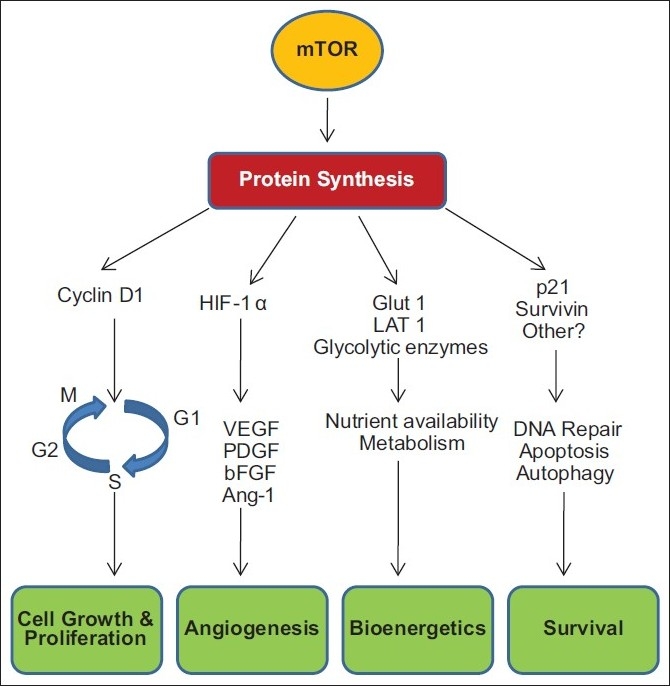
mTOR activation supports cancer cell survival

A recent study has shown that mTOR activation of pS6K increases protein levels of survivin, an inhibitor of apoptosis protein (IAP). Survivin blocks extrinisic and intrinsic apoptotic pathways, and survivin expression has been observed in a wide number of tumor types and associated with poor prognosis in cancer [Figure 1].[16]

Thus, overactivation of mTOR due to dysregulation of upstream pathways, leading to abnormal activities in cell progression, angiogenesis, cell metabolism and apoptosis has been implicated in various cancer types.

## RENAL CELL CARCINOMA

mTOR controls production of HIF-1α, an important protein in RCC, where its unregulated activity is causally related to disease pathogenesis.[17] mTOR regulates the production of several angiogenic growth factors in RCC. mTOR may control the ability of neovascular cells to respond to growth factors.[13,18] mTOR controls cell growth and cell division in RCC and in cells of the tumor microvasculature, and is often dysregulated in renal cancer by signaling defects upstream of mTOR in the PI3K/Akt/mTOR pathway.[19] mTOR regulates nutrient uptake and cell metabolism and contributes to the characteristic metabolic changes in RCC.[1]

## NEUROENDOCRINE TUMOR

Several important molecular changes in NETs involve the mTOR pathway. Increased growth factor signaling, namely, epidermal growth factor (EGF) and insulin-like growth factor (IGF) signaling upstream of mTOR, has been observed frequently in NETs.[20] Also, insulin secretion is believed to be involved in the autocrine activation of mTOR in pancreatic beta cell tumors.[21] mTOR is activated by many gene mutations associated with NETs (germline deletion of the VHL gene).[12] mTOR directs the supply of nutrients to cancer cells by regulating angiogenesis. NETs are highly vascular.[22] VEGF expression has been observed in 80–86% of gastrointestinal carcinoid and pancreatic islet cell tumors.[23]

## GASTRIC CANCER

mTOR is activated in 60–80% of gastric adenocarcinomas[24,25] and is expressed in early-stage and advanced-stage disease, in both diffuse and intestinal subtypes, and in tumor cells that invade lymphatic channels. The mTOR pathway is activated by multiple growth factor receptors, namely, epidermal growth factor receptor (EGFR), Human Epidermal growth factor Receptor 2 (HER2), insulin-like growth factor type 1 receptor (IGF-1R), that are overexpressed in many gastric tumors.[26,27] mTOR regulates production of angiogenic factors (VEGF/VEGFR) that promote new vessel formation and predict poor outcome in patients with gastric cancer.[28] mTOR regulates nutrient uptake and cell metabolism and contributes to the characteristic metabolic changes in cancer. HIF-1α is expressed in most gastric cancers, and HIF-1α expression at the invading tumor edge is associated with advanced-stage disease, lymph node metastases, and poor survival.[29,30]

## BREAST CANCER

mTOR signaling is critical in the pathogenesis of breast cancer. mTOR signaling may be related to estrogen receptor (ER) activation and adaptive estrogen hypersensitivity.[31] mTOR pathway signaling is increased in HER2+ tumor cells resistant to endocrine therapy.[32] mTOR activation predicts a worse clinical outcome for patients treated with endocrine therapy.[33] mTOR controls the supply of nutrients to cancer cells by regulating nutrient uptake, cell metabolism, and angiogenesis.

## HEPATOCELLULAR CARCINOMA

mTOR-dependent signaling is active in 25–45% of hepatocellular carcinoma (HCC).[34] Activation correlates with shorter overall survival; mTOR activation is an independent predictor of recurrence after surgery.[35] mTOR regulates production of angiogenic factors. High VEGF levels have been associated with tumor cell proliferation, poor encapsulation of the tumor nodules, venous invasion, higher grade, and a poor prognosis following resection.[36] mTOR activation through PI3K/Akt pathway is associated with increased expression of growth factors such as EGF, TGF-α, IGF, and hepatocyte growth factor (HGF) that promote HCC cell proliferation and survival.[37,38]

## LYMPHOMA

Approximately 85% of Non-Hodgkin lymphoma (NHL) s arise from cells of B-cell lineage.[39] mTOR signaling is activated in Hodgkin lymphoma cell lines and primary tumors.[40] Cyclin D1 overexpression is a characteristic feature of mantle cell lymphomas.[41] PI3K and Akt overexpression is frequently observed in several B-cell lymphomas.[42]

## CLINICAL RELEVANCE OF mTOR INHIBITION IN CANCER THERAPY

Because the number of potential defects that can cause inappropriate activation of mTOR is large and one or the other is common to most cancer cells, blocking their effect at the point of convergence is a rational approach. The mTOR protein itself is seldom altered, suggesting that mTOR is a stable target for influencing several important pathways in cancer.[43] Furthermore, identifying these molecular defects in a tumor may provide the biomarkers that determine whether the cancer will be sensitive to mTOR inhibition and help select the most appropriate treatment strategy.

Thus far, clinical studies have supported preclinical findings regarding the importance of mTOR in cancer and validate mTOR inhibition as an effective cancer therapy. Given that the mTOR pathway is deregulated in a number of cancers, it is anticipated that mTOR inhibitors will have broad therapeutic application across many tumor types. It is possible that only a subset of cancer patients will have tumors sensitive to mTOR inhibitors as a monotherapy. Based on preclinical findings, mTOR inhibitors may be more efficacious when used in rational combination with other cancer regimens with activities supplemental to and/or influenced by mTOR activity, such as DNA-damaging and hormonal agents, oncogene inhibitors, and other targeted therapies. Thus, mTOR inhibitors may play an important role in the management of cancer. mTOR inhibition has been proven to result in reduced tumor cell growth and proliferation, decreased tumor angiogenesis and inhibition of cell metabolism.

Treatment of advanced RCC has evolved rapidly in the last few years. The emergence of multiple new treatments has led to frequent use of sequential VEGF tyrosine kinase inhibitor (TKI) therapy. Until now, no treatment demonstrated Phase III evidence after disease progression on an initial VEGF-targeted therapy. Everolimus is the first and only therapy with Phase III evidence after failure of VEGF-targeted therapy. Everolimus is a once-daily oral inhibitor of mTOR indicated for the treatment of advanced RCC in patients whose disease has progressed on or after treatment with VEGF-targeted therapy.

## CONCLUSION

mTOR inhibitors are being investigated in clinical trials in several hematologic, gastrointestinal, genitourinary, and neurologic cancers, NETs, and sarcomas, as well as in breast and lung cancers. It is hoped that with this added knowledge, the potential beneficial effects of inhibiting mTOR signaling in cancer patients will be fully recognized and realized in clinical practice.
